# *“Xylella is the Enemy that Must be Fought”*: Representations of the *X. Fastidiosa* Bacterium in the Media Discourse

**DOI:** 10.1007/s41701-022-00129-4

**Published:** 2022-11-02

**Authors:** Tijana Vesić Pavlović, Danijela Đorđević

**Affiliations:** 1 University of Belgrade, Faculty of Mechanical Engineering, Department of Industrial Engineering, Cabinet of Foreign Languages, Belgrade, Serbia; 2 University of Belgrade, Faculty of Agriculture, Cabinet of Foreign Languages, Belgrade, Serbia

**Keywords:** *Xylella fastidiosa*, public discourse, media representations, conceptual metaphors, English

## Abstract

The paper explores media representations of *Xylella fastidiosa*, the bacterium that causes severe plant diseases, using data from online sources in English which reported on the developments in the bacterium spreading and treatment from 2015 to 2020. Two directions of analysis are pursued, the quantitative and qualitative one. Quantitative analysis reveals that the bacterium and its effects are described via the terms belonging to the lexical fields of fear, diseases, change, the supernatural, hostility, destruction, killing and war, with the latter three being the most dominant statistically speaking. Further, qualitative analysis attests that some of these terms are used metaphorically as instantiations of the war metaphor, which is generally effective in communicating the severity of the *X. fastidiosa* induced diseases and mobilising the necessary support. Based on the results, it may be argued that the language used in the media for describing *X. fastidiosa* fits into the prevalent “catastrophe discourse”, with the purpose of raising awareness of the gravity of the threat the bacterium poses, as well as justifying the severe measures undertaken to contain it.

**Supplementary Information:**

The online version contains supplementary material available at 10.1007/s41701-022-00129-4.

## Introduction

Although relatively unknown to a wider public, the bacterium *Xylella fastidiosa* has spurred much scientific concern in the affected European countries, as well as in those areas where it might yet occur. *X. fastidiosa* is a gram-negative xylem-limited fastidious bacterium (Wells et al., 1987, p. 149), an insect-transmitted plant pathogen which colonises the xylem of around 600 host plant species (European Food Safety Authority, 2020). Starting from the 19th century, it has affected vineyards in California, and later it emerged in Brazil, causing citrus diseases (Sicard et al., 2018). The first report of *X. fastidiosa* under field conditions in the European Union was in late 2013 when it was discovered in southern Italy, in the Lecce province in Apulia (Saponari et al., 2013). The disease consequently spread to parts of France, Spain and Portugal, mainly striking olive trees, but also other plants such as oleander, almond, cherry, many ornamentals and endemic Mediterranean species (Saponari et al., 2019). The infected plants exhibited a complex of very grave symptoms, such as leaf scorch and marginal necrosis, chlorosis and wilting, collectively known as the olive quick decline syndrome (OQDS) (Martelli et al., 2016).

Due to its severe impact on the plants, the actions undertaken to contain the spread of the bacterium in Italy included eradication measures, i.e. destroying the infected trees, followed by containment measures, which implied that even the healthy trees needed to be removed within the radius of 100 m of the infected ones, forming the so-called ‘buffer zone’ (Saponari et al., 2019, p. 179). This was met with the opposition from local people, growers and other stakeholders (Saponari et al., 2019) since the olive tree represents their heritage and tradition (Ali et al., 2020). Further, people started making up different conspiracy theories that obstructed interventions, monitoring and management strategies (Saponari et al., 2019), making it difficult to deal with the spread of the disease (Almeida, 2016). Some of the olive growers and activists did not accept that trees were infected (Nadeau, 2015) and even accused nine researchers of potential involvement in the disease outbreak (Abbott, 2015). Although researchers were later officially acquitted of such allegations, some social movements started proposing their own solutions, alternative to those prescribed by scientists, which relied on pruning, application of fungicides and organic fertilisers instead of complete eradication of the trees (Colella et al., 2019). To date, *X. fastidiosa* associated diseases remain incurable, but for eradication and containment measures used to limit and eliminate the pathogen (Saponari et al., 2019).

The purpose of this paper is to explore how *X. fastidiosa* is represented in the media sources available online in the English language. Numerous previous studies on disease discourses have discovered various language patterns employed in communicating the effects of grave diseases such as the foot and mouth disease (Nerlich et al., 2002; Nerlich & Döring, 2005; Nerlich, 2004, 2007, 2011), severe acute respiratory syndrome (SARS) (Wallis & Nerlich, 2005), avian flu (Nerlich & Halliday, 2007; Koteyko et al., 2008a), the methicillin-resistant *Staphyloccous aureus* (Crawford et al., 2008; Koteyko, Nehrlich et al. 2008; Washer & Joffe, 2006) and multi-drug resistant bacteria (Peters et al., 2019), and, as of late, the COVID-19 pandemic (e.g. Chaiuk & Dunaievska, 2020; Katermina & Yachenko, 2020). They have found that military language seems to be prevalent in disease discourses, specifically when it comes to describing the epidemics of viruses and bacteria, which has led some researchers to speak of the dominance of “catastrophe discourse” in describing diseases (Nerlich & James 2009).

To our knowledge, no study has yet explored the representation of *X. fastidiosa* in public discourse and this is the focus of the current paper. Hence, we set two main research aims in the paper: (1) to identify the main lexical fields present in describing the *X. fastidiosa* bacterium; and (2) to analyse the identified instances of use of metaphorical language in describing the *X. fastidiosa* bacterium. The study is conducted on the material collected from mainstream media sources in English which covered the developments regarding the spread of the bacterium throughout Europe in the period from 2015 to 2020. We use the methods of corpus linguistics to identify the most frequent lexemes in the analysed material used for describing the bacterium, as well as conceptual metaphor analysis to explore the most prevalent conceptual metaphor present in *X. fastidiosa* framing, namely, the war metaphor.

The paper is organised as follows. In the second section, we elaborate on the findings of the previous studies of disease discourses. Section 3 contains information about the used methodology and materials, while Sect. 4 provides an overview of research results – the results of quantitative analysis are given in the first subsection and the second subsection specifically dwells on the instances of identified metaphoric language. We conclude with a brief summary of the findings and possible directions for further research.

## Previous Studies

Various studies dealt with the framing of different diseases in the public discourse and their impact upon the implemented policies, mostly using the English language material.

In their analysis of the framing of the foot and mouth disease (FMD), Nerlich et al. (2002) found that the conceptualisation of FMD relied on several narratives (such as war, contest, journey or plague), different images and metaphors. The image of an enemy in war was central, followed by “a rival in a fight, a rival engaged in a contest or race with humans, a criminal victimising humans, an evil and mysterious entity and a plague or even death itself” (Nerlich et al., 2002, p. 95). The actions taken against FMD were conceptualised in terms of fighting a war against it, so FMD had to be *combated*, *eradicated*, *defeated*, *exterminated*, *wiped out* etc. (Nerlich et al., 2002, pp. 99−100). In this case, using well-understood source domains to cope with FMD “helped create a ‘common ground’ for communication between the media, the public, and policy makers” (Nerlich et al., 2002, p. 93).

SARS virus was framed in similar terms in the media, dominantly as a killer (e.g. *a killer virus*, *claiming victims*) which inspired fear in a supernatural manner (Wallis & Nerlich, 2005). The other used metaphor was that of control, which referred to the response to the disease. Interestingly, usually ubiquitous in the representation of diseases, war metaphors were missing from the SARS coverage (Wallis & Nerlich, 2005). In the case of avian flu, the metaphors based on the journey and war scenarios were also present, such as the spread of a virus is a journey and the appearance of a virus is an invasion (Nerlich & Halliday, 2007; Koteyko et al., 2008a).

The bacterium which has gained a great deal of attention in terms of its framing in public discourse is the methicillin-resistant *Staphyloccous aureus* (MRSA), which was labelled superbug^1^ due to its invincibility (Washer & Joffe, 2006, p. 2148). Various scenarios and metaphors were used in the UK press coverage of MRSA in a ten-year period (Crawford et al., 2008). At first, the dominant scenarios included those of war, fight and battle, depicting the doctors losing the battle with the bacterium and the bacteria *invading*, *colonising* and *spreading* (Crawford et al., 2008, p. 336). In 2000s, these scenarios were mostly replaced by those of crime, struggle and contest, while at the end of the period, in 2005, the most prevalent was the metaphor of a matron and the image of cleanliness. These changes in metaphor scenarios actually implied changes in the interpretations of the causes for the MRSA rise.

The afore-described studies revealed a number of narratives, scenarios and conceptual metaphors used in the public discourse on specific diseases, at times even establishing a connection between the specific framing of diseases in public discourse and the policies undertaken to deal with them. In our analysis of the representations of *X. fastidiosa* in public discourse, we seek, among other things, to compare the findings obtained on the collected corpus of articles in English about *X. fastidiosa* with those yielded by the above-presented research.

## Materials and Methods

The corpus for the current analysis was compiled from online sources (websites and print media webpages) in English which reported on the developments in the *X. fastidiosa* spread and control. It was necessary to include a number of available sources, firstly, since the disease is not contained to one country and of relevance to particular media only, and secondly, because reporting on *X. fastidiosa* is scarce in mainstream media in English. These websites were chosen because they are readily accessible, reach many people and also provide a coverage of other important global issues. Hence, we deemed the analysis of their reporting on the topic of *X. fastidiosa* important since it may offer insight into the patterns of communicating about the disease not only with the interested parties, but with the general public as well, in the English language which is considered as a lingua franca in the modern world.

We first selected the websites of media sources in English which are usually used in the analyses of this type (e.g. *The Guardian*, *Daily Mail*, *The Independent*, *The Telegraph* and the *BBC*). Next, we chose to include one particular website, *Olive Oil Times*, since the web search proved it provided extensive coverage of the *X. fastidiosa* issue. Then we searched for the texts on these websites^2^ which contained the key word *Xylella fastidiosa*. The search was restricted to the period from 2015 to 2020 since it marks the period of intensive activity of the bacterium in Europe.

The collection of texts obtained in this way comprised 150 texts (totalling 82,611 words) that were published in English only in the afore-said period. The majority of texts (n = 76; 40,261 words) came from the website *Olive Oil Times*. The collected texts mainly describe the developments in Italy, France and Spain pertaining to *X. fastidiosa* progress and treatment in the afore-mentioned period. They comment on the uniqueness of the bacterium, its disastrous impact on plants (most notably, olive trees, but also other trees), the progress of the bacterium across the Mediterranean countries and the actions that the governments of these countries undertook in order to eradicate it. The British sources also focus on the possible consequences of *X. fastidiosa* being found in Britain and the ways to prevent this.

First, the collected texts were carefully read, and all the instances in which the bacterium was discussed in a certain way were marked by both authors. Then, through joint discussion, we compiled a list of topics pertaining to the *X. fastidiosa* that were discussed and subsumed them into one of three categories – descriptions of the bacterium, descriptions of its effects and descriptions of the actions taken against it. The lexemes occurring in each of these categories were selected. It was noticed that these lexemes could be grouped into particular lexical fields and that they were used both literally and metaphorically. In the next step, we focused on these particular lexemes and performed a quantitative analysis pertaining to the frequency of their occurrence so as to discover the prevalence of certain lexical fields^3^ in the descriptions of *X. fastidiosa* in the selected texts^4^. For this purpose we employed the #LancsBox software (Brezina et al., 2015, 2018, 2020), more precisely, the KWIC tool, to find and count all instances of the lexemes searched. This facilitated counting the lexeme occurrences, as well as checking the context of their occurrence. The method of using quantitative corpus analysis and investigating lexical fields occurring in the metaphors in discourse on marketing was previously conducted by Koller (2004, pp. 65−78).^5^

Finally, we analysed the cases where the lexemes were used metaphorically according to the tenets of conceptual metaphor theory and critical discourse analysis (Charteris-Black & Musolff, 2003; Charteris-Black, 2004), using the MIP procedure (Pragglejaz, 2007)^6^.

## Results

### Major Lexical Fields in the Descriptions of *X. Fastidiosa* in the Analysed Texts

Table 1 shows the lemmas^7^ occurring in the descriptions of *X. fastidiosa* in the analysed corpus and their semantic fields. Absolute frequencies of occurrence of these lemmas as well as the normalised frequency of occurrence per 1,000 words are provided. We found lexemes dominantly belonging to several lexical fields such as fear, destruction, killing and war, as well as other diseases, the supernatural, hostility and change.

**Table 1:** The identified lemmas in descriptions of the bacterium classified according to semantic fields

Lexical fields and lemmas	AF	NF
**Fear**		
*fear*	37	0.45
*dread*	4	0.05
**Disease**		
*Ebola*	11	0.13
*leprosy*	7	0.08
*plague*	5	0.06
**Change**		
*game changer*	5	0.06
**Supernatural**		
*ominous*	3	0.04
*nightmarish*	1	0.01
**Hostility**		
*vicious*	2	0.02
*hostile*	1	0.01
*merciless*	1	0.01
*aggressive*	1	0.01

Note: AF – absolute frequency, NF – normal frequency

The most frequently used lemmas in the *X. fastidiosa* descriptions come from the semantic field of fear – the bacterium is described as *the dreaded olive tree killer* or *one of the most feared plant pathogens in the world*. *X. fastidiosa* is also compared to other, more familiar perilous diseases and called *olive Ebola*, *the Ebola of olive trees*, *olive tree leprosy* or *a lethal plague*. The most frequent of these is *Ebola* (n = 11; 0.13 occurrences per 1,000 words), followed by *leprosy* (n = 7; 0.08) and *plague* (n = 5; 0.06). The bacterium is also described as *a game changer* or *a game changing plant disease*, which emphasises its fairly unique or unprecedented nature. Adjectives that occur in the bacterium descriptions are dominantly negative – coming from the field of the supernatural (“*an ominous plant disease*” [OOT^8^, 04/05/18], which produces “*the strange and nightmarish result*” [OOT, 07/07/17]) or hostility (a *vicious bacterium*, *hostile species*, *aggressive pathogen*, *merciless disease*).

Based on our analysis, it could be inferred that, in describing the effects of the bacterium, media discourse on *X. fastidiosa* relies on the lexical field of destruction and killing (Table 2).

**Table 2:** The identified lemmas in the descriptions of the effects of *X. fastidiosa* classified according to semantic fields

Lexical fields and lemmas	AF	NF
**Destruction**		
*destroy*	67	0.81
*devastate*	67	0.81
*damage*	42	0.51
*wipe out*	32	0.39
*ravage*	18	0.22
*decimate*	8	0.10
*wreak havoc*	4	0.05
**Killing**		
*die*	111	1.34
*kill*	53	0.64
*lethal*	6	0.07
*fatal*	3	0.04
*strangle*	3	0.04
*starve*	1	0.01

Note: AF – absolute frequency, NF – normal frequency

A variety of verbs is used to describe the destruction *Xylella* creates: it *damages* the plantations, *blights* the crops, *rips through* olive groves, may *devastate* or *ravage* trees, with the highest frequencies of the lemmas *destroy*, *devastate*, *damage* and *wipe out* (Table 2). Its potential for destruction is described as enormous, both in terms of the time needed to destroy the plants (e.g., it has “*the ability to destroy multiple crops in rapid fashion*” [OOT, 07/07/17]) and the number of plants it can destroy (e.g. it “*has been responsible for the destruction of hundreds of thousands of acres of olive trees*” [OOT, 5/04/19]). It is uncontrollable, so it *runs rampant* across olive plantations in Southern Italy or *wreaks havoc* on Southern France’s olive trees. In certain cases, verbs from this field are used metaphorically – *X. fastidiosa* can destroy the whole olive oil industry and even culture and traditions of the affected country (“*centuries of history, culture and traditions are destroyed*” [OOT, 12/09/19]).

Another highly frequent group of lexemes used to describe the effect of *X. fastidiosa* on plants in the analysed texts belongs to the lexical field of killing (Table 2). The lemma which features most prominently with this respect is *die* (n = 111; 1.34). Plants usually suffer death as a consequence of contact with the bacterium, which is evident in its descriptions as *a tree-killing bacterium*, an *olive killer disease*, *the olive tree killing XF epidemic*, *killer bacteria*, or the fact that it is fatal to its victims (*deadly*, *fatal*, *a lethal plant bacterium* or a *lethal olive disease*). This killer has a vast killing potential in terms of the number of plant species it can kill – sources mention that it can kill “*over 200 types of plant*”. *X. fastidiosa* may also be deemed to be a mass murderer since it has *killed a million olive trees in Italy* and continues *to kill tens of thousands of olive trees*. The specific MO of this killer is either strangulation (“*the same deadly plant pathogen strangling trees farther to the south*” [OOT 20/11/17]) or starvation (“*a virulent pathogen that starves olive trees*” [BBC 9/01/15]).

Lexemes from the lexical field of war are used to describe the actions undertaken to stop the spreading of *X. fastidiosa* (Table 3), with the dominantly used lemmas *fight*, *combat*, *attack* and *invade*. This implies that, like many other diseases or pests described within various discourses, *X. fastidiosa* is powerful and evasive, thus difficult to tackle.

**Table 3:** The identified lemmas in the descriptions of the actions taken against *X. fastidiosa* − the lexical field ‘war’

Lexical field and lemmas	AF	NF
War		
*buffer zone*	49	0.59
*fight*	29	0.35
*combat*	23	0.28
*attack*	22	0.27
*invade*	18	0.22
*defend*	12	0.14
*battle*	9	0.11
*arm*	5	0.06
*war*	2	0.02
*colonize*	2	0.02
*enemy*	1	0.01
*victory*	1	0.01
*weapons*	1	0.01

Note: AF – absolute frequency, NF – normal frequency

All these lexemes are used metaphorically within the broader frame of the war metaphor, which, in this case, can be formulated as trying to stop the spread of X. fastidiosa is waging a war. We further elaborate on the instantiations of this metaphor in the analysed set of texts in the following subsection.

### Instantiations of the war Metaphor in Describing the Actions Taken against *X. Fastidiosa*

Numerous studies have emphasised the ubiquity of war metaphors in public discourse for framing various issues, e.g. in the language about invasive species (Larson, 2005), in medicine (Mongoven, 2006; Segal, 1997) or in disease discourses (Nerlich et al., 2002). The prevalence of war metaphors may be attributed to the fact that they convey a sense of urgency, and are thus suitable for framing adversarial relationships (Flusberg et al., 2018, p. 4–5). The obvious benefit is that “the language of war can help people recognise the threat that diseases pose to public health” (Flusberg et al., 2018, p. 6). Still, there are numerous downsides pointed by different authors, for instance, that war metaphors may have possibly become too ubiquitous and that they are used for insignificant issues as well, which may reduce their strength (Flusberg et al., 2018, p. 9).

In the analysed texts, the EU countries, scientists and growers wage the war against *X. fastidiosa*. Since *X. fastidiosa* is an *enemy* that needs to be defeated, countries are called upon to *fight*, *battle* or *combat* the bacterium. The war against the enemy involves forming alliances between stakeholders or countries in order to ensure victories (“*Europe joins forces to fight Xylella fastidiosa*” [OOT 29/12/16]). Country officials express a resolute attitude in this fight: “*We are facing this plague with seriousness and determination*” (OOT 23/09/19).

As a true soldier, *X. fastidiosa* is *marching*, and its march is deadly (“*this quiet corner of Puglia is now the northern tip of the deadly march of XF*” [OOT 20/11/17]). It *attacks* olive groves or vines. *Xylella* either invades plants and their parts (“*the pathogen colonizes the vessels that a plant uses to transport water and nutrients*” [OOT 27/11/16]) or whole countries and areas (“*the disease has invaded 23000 hectares in Puglia*” [OOT 20/11/17]). This is consistent with the framing of another disease, SARS, in terms of the metaphor, the appearance of a virus is an invasion (Nerlich & Halliday, 2007).

The battlefields in the war against *X. fastidiosa* are vividly depicted, containing front-lines, war zones and buffer zones established to stop the enemy’s advances (ex. 1, 2, 3)^9^:

(1) *Only 20 km south from the green hills of the Valle d’Itria, groves near the town of Oria are under attack. In the space of two years, groves here have become a kind of war zone – a scene out of a picture book on plagues*. (OOT 20/11/17)

(2) *Where the Olive Trees Are Dying: A Front-Line Report on Xylella* (OOT 20/11/17).

(3) […] *there is no effective treatment for infected plants and commission regulations say the only solution is to destroy them and establish Xylella-free buffer zones around them* […]. (BBC 19/12/15)

The sources state there has been no major success yet in winning the war against the bacterium and reports reflect on the difficulties in stopping the enemy from advancing (e.g. the bacterium *is* “*spreading ‘inexorably’ north*”). Even the victories in the war bring about a sense of caution that the battle against *X. fastidiosa* is nowhere near its end (ex. 4).

(4) *Andalusia declares its agriculture Xylella-free […] so far so good for Andalusia though the battle against the bacterium is certainly far from over […]*. (OOT 01/04/16)

Farmers express a very pessimistic attitude of defeat in the battle against *X. fastidiosa*, feeling like inept soldiers without the appropriate weapons to fight it (“*no weapons with which to fight it back*” [DM 16/02/2016]). This creates the sense of having lost the war against the bacterium, which will result in disastrous consequences of the enemy spreading further and capturing more territories (ex. 5).

(5) *Now, five years into the tragedy, it’s becoming ever more ominous that the battle to eradicate Xylella may have been lost and scientists fear its spread may now be unstoppable and potentially even spread faster*. (OOT 04/05/18)

The results of the battles against *X. fastidiosa* even prompt reflections of the need to learn how to live with the enemy since a complete victory currently seems unlikely: “*the reduction of the bacterium is not impossible – co-existence is something that can be achieved*” (OOT 09/08/18).

In line with previous considerations, the use of various facets of war metaphor for the framing of actions against the spread of *X. fastidiosa* serves to underscore the gravity of the disease and communicate the urgency and resoluteness with which the issue needs to be addressed. However, in this specific case, the communicated sense of urgency of waging war against *X. fastidiosa* and enforcing drastic actions against it started a war of another kind. As mentioned earlier, the actions for fighting *X. fastidiosa* in a certain area implied the destruction of the affected trees, but also the healthy ones surrounding them within a certain radius. This kind of measures led to protests among the farmers who suffered most from these decisions, particularly those in Italy. The measures were perceived as a “*condemnation of death to the territory*” (OOT 23/09/15). Thus, judging by the examples from the analysed material, it may be argued that growers started to see themselves as soldiers who should wage war against the drastic measures, for example, fight the “*battle for the survival of Salento*” (OOT 23/09/15), the area which was amongst the most heavily affected by the uprooting of trees (ex. 7). Hence, the authorities actually had to remind the farmers that “*Xylella is the enemy that must be fought*” (OOT 14/06/18).

It is evident that the use of the war metaphor in the analysed public discourse about *X. fastidiosa* served to justify the drastic policies for its eradication, but also that the same conceptual metaphor underlay the discourse of the farmers who opposed the measures and thought of themselves as waging the war against the very authorities who imposed the said measures. As in the case of the discourse on the previously studied diseases, this indicates the need for considering the positions of all the stakeholders involved in such a complex issue such as *X. fastidiosa*, so as not to cause the gap in communication between scientists and growers.

## Conclusion

Given its impact on agriculture not only in European countries, but globally, this research focused on media depictions of the bacterium *Xylella fastidiosa*, the pathogen that causes severe plant diseases, using data from online English sources that reported on the bacterium spreading and treatments from 2015 to 2020. The quantitative and qualitative approaches to analysis were employed. Quantitative analysis shows that the bacterium and its effects are mostly characterised using lexemes from the lexical fields of fear, diseases, change, the supernatural, hostility, destruction, killing and war. Statistically speaking, the most dominant fields are those of destruction (n = 238; 2.89), killing (n = 177; 2.14) and war (n = 174; 2.11), which implies that the lexemes belonging to these fields were used much more frequently to describe the characteristics of *X. fastidiosa* and its effects, as well as the actions aimed against it, than, for instance, lexemes from the fields of fear (n = 41; 0.50), diseases (n = 23; 0.27), change (n = 5; 0.06).

Further qualitative analysis has attested that some of the phrases within these lexical fields, mostly in the lexical field of war, are employed metaphorically as instantiations of the war metaphor.

Since many things about the spread of the bacterium are still not known, it is perceived as something unfathomable, evasive and intangible, which is attested by frequent use of the lexemes from the semantic fields of fear and supernatural. Further, the comparison with the familiar devastating diseases (Ebola, leprosy, plague) should raise awareness about the significance of the threat *X. fastidiosa* poses for the affected species. The registered frequent use of lexemes from the semantic fields of destruction and killing additionally underscores the need to fear the impenetrable vicious creature that destroys trees in the cruellest and most horrid manner.

The actions taken against the bacterium are described in terms of waging a war against it, which entails being engaged in a battle, defeating the enemy or being defeated. Since *X. fastidiosa* is so formidable, it is implied that only the war against it could work, not more peaceful methods, thus the dominance of the war metaphor in framing the response to the disease.

Based on the afore-presented data analysis results, it may be argued that there is a similar pattern in the representation of *X. fastidiosa* as in the previously described studies of the framing of other epidemic diseases, most notably the FMD, MRSA and the SARS virus. This refers to *X. fastidiosa* being described as other disease or a supernatural force (Nerlich et al., 2002). It is a killer bacterium (similar to the killer SARS virus in Wallis and Nerlich, 2005); it also includes the ‘epidemics as nightmares’ framing (Nerlich et al., 2002) and resorting to war metaphors in describing the actions necessary to deal with it (Nerlich & Halliday, 2007). As of late, the first analyses of the coverage of the current pandemic of COVID-19 have shown that, among other things, war and military metaphors were utilised to a great extent (e.g. Nerlich, 2020; Sabucedo et al., 2020), particularly to justify the strict measures introduced to stop the spreading of the virus (Gillis, 2020), which has resulted in the appeal to reframe the metaphors and use more suitable ones, for instance, the fire metaphor (Semino, 2020).

In conclusion, it can be stated that the main purpose of relying on the lemmas from the semantic fields in communication on the bacterium *X. fastidiosa* spreading and effects is to raise awareness of the gravity of its occurrence, as well as to instil fear in all the parties involved to take the matter seriously and deal with it accordingly. Bearing in mind previous studies that found that apocalyptic scenarios were common in disease discourses, based on the results obtained in this study, it may be argued that the language used in the media for describing *X. fastidiosa* fits into the prevalent “catastrophe discourse”,

Additionally, the employment of the military and war metaphors serves to justify the radical treatment of the trees affected by *X. fastidiosa* (uprooting both them and the surrounding trees), which has been staunchly opposed by one portion of the olive farmers so far. However, as demonstrated previously in the pertinent literature, the imposition of one metaphor upon a certain issue may lead to the acceptance of only one way to deal with the disease, which, in turn, may cause a revolt among those affected. This has been mentioned as a frequent issue in political discourse, namely, “if public political debate implements one given metaphor again and again – then that metaphor becomes our primary way of perceiving issue at hand” (Lakoff & Wehling, 2016, p. 24). It may be argued that the war metaphor is rather effective in raising awareness about the severity of the *X. fastidiosa* induced diseases and mobilising the necessary support; still, our findings indicate that this narrative is used in another way. Namely, the farmers affected by the strict measures also used the war narrative but spoke of a different enemy – the measures themselves. The case of *X. fastidiosa* may thus serve as a warning against the automatic use of war metaphors in framing the actions against the disease because it simply cannot justify any policy aimed at eradicating the disease, quite the opposite.

The present study is fairly limited in scope since it was conducted on a specific set of texts available through Internet search and in the English language only. Hence, future research might focus on analysing the texts in other languages, most dominantly, Italian and Spanish, since these country were among the most severely affected by *X. fastidiosa*. Another direction for further studies would be a comprehensive analysis of the semantic fields and metaphors employed in the specific discourses of different stakeholders involved in the *X. fastidiosa* issue (officials, scientists, growers) and the materials used to communicate with them about the disease in order to determine how the used language patterns shape their understanding of the serious problem posed by the bacterium *X. fastidiosa*.

## Electronic Supplementary Material

Below is the link to the electronic supplementary material.

Supplementary Material 1

## Appendix

### The lexical field of fear

**Table:**

Noun	Verb	Adjective
-	to dread	dreadful
fear	to fear	-

### The lexical field of disease

**Table:**

Noun
Ebola
leprosy
plague

### The lexical field of change

**Table:**

Noun	Verb	Adjective
game changer	-	game changing

### The lexical field of the supernatural

**Table:**

Noun	Verb	Adjective
-	-	ominous
-	-	nightmarish

### The lexical field of hostility

**Table:**

Noun	Verb	Adjective
-	-	vicious
-	-	hostile
-	-	merciless
-	-	aggressive

### The lexical field of destruction

**Table:**

Noun	Verb	Adjective
damage	to damage	-
-	to destroy	destructive
devastation	to devastate	devastating
-	to decimate	-
-	to ravage	-
-	to run rampant	-
-	to wipe out	-
-	to wreak havoc	-

### The lexical field of killing

**Table:**

Noun	Verb	Adjective
death	to die	deadly
-	-	fatal
killer	to kill	-
-	-	lethal
-	to strangle	-
-	to starve	-

### The lexical field of war

**Table:**

Noun	Verb	Adjective
army, arms	-	-
attack	to attack	-
battle	to battle	-
buffer zone	-	-
defence	to defend	defensive
-	to colonize	-
-	to combat	-
enemy	-	-
fight	to fight	-
invader, invasion	to invade	invasive
victory	-	-
war	-	-
weapon	-	-

## Footnotes

1 Similarly, there is an absorbing study on the “superweed” Palmer amaranth (Bétrisey et al., 2021), providing an analysis of metaphors used in various discourses (including different genres and styles) to build frames for justifying aggressive eradication measures of this inimical plant.

2 The following websites were searched: https://www.bbc.com/, https://www.dailymail.co.uk, https://www.theguardian.com/uk, https://www.independent.co.uk, https://www.oliveoiltimes.com/ and https://www.telegraph.co.uk.

3 The list of fields is by no means exhaustive; we focused on the main perceived patterns of communication about *Xf* instead of noting all minor fields to which other lexemes might have belonged to.

4 Benefits of applying corpus methodology in semantic analyses (e.g. for comparison of the frequency, productivity and creative use of metaphors) have been discussed previously by Oster (2010).

5 Koller investigated metaphorical framing of the field of marketing through the analysis of absolute and relative frequencies of the occurrence of lexemes from different word classes (nouns, verbs and adjectives) belonging to different lexical fields (war, sports, games, romance).

6 This method entails several steps: first, the entire text is read and lexical units determined; the meaning of each unit is then established in context and it is determined whether the unit has a “more basic contemporary meaning” than the meaning in the context at hand; next, it is determined whether the contextual meaning “contrasts with the basic meaning but can be understood in comparison with it”; if the answer is affirmative, the lexical unit is considered metaphorical (Pragglejaz, 2007, p. 3).

7 All the identified lemmas within different lexical fields are shown in the Appendix.

8 Abbreviations that refer to the specific source are the following: BBC: British Broadcasting Corporation, DM: Daily Mail, OOT: Olive Oil Times.

9 The symbol […] indicates that parts of the example have been omitted.

## References

[CR1] Abbott A (2015). Scientists blamed for olive-tree ruin: Italian police investigate researchers’ role in a bacterial epidemic that is devastating Puglia’s olive groves. Nature.

[CR2] Ali BM, van der Werf W, Lansink AO (2020). Assessment of the environmental impacts of *Xylella fastidiosa* subsp. *pauca* in Puglia. Crop Protection.

[CR3] Almeida RPP (2016). Can Apulia’s olive trees be saved?. Science.

[CR4] Bétrisey F, Boisvert V, Sumberg J (2021). Superweed amaranth: metaphor and the power of a threatening discourse. Agriculture and Human Values.

[CR5] Brezina V, McEnery T, Wattam S (2015). Collocations in context: A new perspective on collocation networks. International Journal of Corpus Linguistics.

[CR6] Brezina, V., Timperley, M., & McEnery, T. (2018). #LancsBox v. 4.x [software]. Available at: http://corpora.lancs.ac.uk/lancsbox

[CR7] Brezina, V., Weill-Tessier, P., & McEnery, A. (2020). #LancsBox v. 5.x. [software]. Available at: http://corpora.lancs.ac.uk/lancsbox

[CR8] Chaiuk TA, Dunaievska OV (2020). Fear culture in media: an examination on coronavirus discourse. Journal of History Culture and Art Research.

[CR9] Charteris-Black J (2004). Corpus approaches to critical metaphor analysis.

[CR10] Charteris-Black J, Musolff A (2003). ‘Battered hero’ or ‘innocent victim’? A comparative study of metaphors for euro trading in British and German financial reporting. English for Specific Purposes.

[CR11] Colella C, Carradore R, Cerroni A (2019). Problem setting and problem solving in the case of olive quick decline syndrome in Apulia, Italy: A sociological approach. Phytopathology.

[CR12] Crawford P, Brown B, Nerlich B, Koteyko N (2008). The ‘moral careers’ of microbes and the rise of the matrons: An analysis of UK national press coverage of methicillin-resistant Staphylococcus aureus (MRSA) 1995–2006. Health Risk & Society.

[CR13] European Food Safety Authority (2020). Update of the *Xylella* spp. host plant database–systematic literature search up to 30 June 2019. EFSA Journal.

[CR14] Flusberg SJ, Matlock T, Thibodeau PH (2018). War metaphors in public discourse. Metaphor and Symbol.

[CR15] Gillis M (2020). Ventilators, missiles, doctors, troops… the justification of legislative responses to COVID-19 through military metaphors. Law and Humanities.

[CR16] Katermina V, Yachenko E (2020). Axiology of COVID-19 as a Linguistic Phenomenon in English Mass Media Discourse. Advances in Journalism and Communication.

[CR17] Koller V (2004). Metaphor and gender in business media discourse: A critical cognitive study.

[CR18] Koteyko N, Brown B, Crawford P (2008). The dead parrot and the dying swan: The role of metaphor scenarios in UK press coverage of avian flu in the UK in 2005–2006. Metaphor and Symbol.

[CR19] Koteyko N, Nerlich B, Crawford P, Wright N (2008). ‘Not rocket science’ or ‘No silver bullet’? Media and Government Discourses about MRSA and Cleanliness. Applied Linguistics.

[CR20] Lakoff G, Wehling E (2016). Your brain’s politics: How the science of mind explains the political divide.

[CR21] Larson BM (2005). The war of the roses: Demilitarizing invasion biology. Frontiers in Ecology and the Environment.

[CR22] Martelli GP, Boscia D, Porcelli F, Saponari M (2016). The olive quick decline syndrome in south-east Italy: a threatening phytosanitary emergency. European Journal of Plant Pathology.

[CR23] Mongoven A (2006). The war on disease and the war on terror: A dangerous metaphorical nexus?. Cambridge Quarterly of Healthcare Ethics.

[CR24] Nadeau BL (2015). The battle of olives. Scientific American.

[CR25] Nerlich B (2004). War on foot and mouth disease in the UK, 2001: Towards a cultural understanding of agriculture. Agriculture and Human Values.

[CR26] Nerlich B (2007). Media, metaphors and modelling: How the UK newspapers reported the epidemiological modelling controversy during the 2001 foot and mouth outbreak. Science Technology & Human Values.

[CR27] Nerlich B, Handl S, Schmid HJ (2011). The role of metaphor scenarios in disease management discourses: Foot and mouth disease and avian influenza. Windows to the Mind: Metaphor, Metonymy and Conceptual Blending.

[CR28] Nerlich, B. (2020). Metaphors in the Time of Coronavirus. Retrieved from https://blogs.nottingham.ac.uk/makingsciencepublic/2020/03/17/metaphors-in-the-time-of-coronavirus/

[CR29] Nerlich B, Döring M (2005). Poetic justice? Rural policy clashes with rural poetry in the 2001 outbreak of foot and mouth disease in the UK. Journal of Rural Studies.

[CR30] Nerlich B, Halliday C (2007). Avian flu: the creation of expectations in the interplay between science and the media. Sociology of Health & Illness.

[CR31] Nerlich B (2009). The post-antibiotic apocalypse” and the “war on superbugs”: catastrophe discourse in microbiology, its rhetorical form and political function. Public Understanding of Science.

[CR32] Nerlich B, Hamilton CA, Rowe V (2002). Conceptualising foot and mouth disease: The socio-cultural role of metaphors, frames and narratives. Metaphorik de.

[CR33] Oster U (2010). Using corpus methodology for semantic and pragmatic analyses: What can corpora tell us about the linguistic expression of emotions?. Cognitive Liguistics.

[CR34] Peters J, Dykes N, Habermann M, Ostgathe C, Heckel M (2019). Metaphors in German newspaper articles on multidrug-resistant bacteria in clinical contexts, 1995–2015: A computer-assisted study. Metaphor and the Social World.

[CR35] Pragglejaz Group (2007). MIP: A method for identifying metaphorically used words in discourse. Metaphor and Symbol.

[CR36] Sabucedo JM, Alzate M, Hur D (2020). COVID-19 and the metaphor of war (COVID-19 y la metáfora de la guerra). International Journal of Social Psychology.

[CR37] Saponari M, Boscia D, Nigro F, Martelli GP (2013). Identification of DNA sequences related to *Xylella fastidiosa* in oleander, almond and olive trees exhibiting leaf scorch symptoms in Apulia (Southern Italy). Journal of Plant Pathology.

[CR38] Saponari M, Giampetruzzi A, Loconsole G, Boscia D, Saldarelli P (2019). *Xylella fastidiosa* in olive in Apulia: Where we stand. Phytopathology.

[CR39] Segal JZ (1997). Public discourse and public policy: Some ways that metaphor constrains health (care). Journal of Medical Humanities.

[CR40] Semino E (2020). “Not Soldiers but Fire-fighters” – Metaphors and Covid-19. Health Communication.

[CR41] Sicard A, Zeilinger RA, Vanhove M, Schartel TE, Beal DJ, Daugherty MP, Almeida R (2018). *Xylella fastidiosa*: Insights into an Emerging Plant Pathogen. Annual Review of Phytopathology.

[CR42] Wallis P, Nerlich B (2005). Disease metaphors in new epidemics: the UK media framing of the 2003 SARS epidemic. Social Science & Medicine.

[CR43] Washer P, Joffe H (2006). The “hospital superbug”: Social representations of MRSA. Social Science and Medicine.

[CR44] Wells JM, Raju BC, Hung HY, Weisburg WG, Mandelco-Paul L, Brenner DJ (1987). *Xylella fastidiosa* gen. nov., sp. nov: Gram-Negative, Xylem-Limited, Fastidious Plant Bacteria Related to *Xanthomonas* spp. International Journal of Systematic Bacteriology.

