# Supplementary material for: “Xylella is the Enemy that Must be Fought”: Representations of the X. Fastidiosa Bacterium in the Media Discourse
Source: Corpus Pragmat. 2022 Nov 2;6(4):291–306. doi: 10.1007/s41701-022-00129-4 (PMC9629884; doi:10.1007/s41701-022-00129-4)
Supplement: Supplementary file 1 — Supplementary Material 1 [file 41701_2022_129_MOESM1_ESM.pdf]

Tijana S. Vesić Pavlović is Assistant Professor of English Language at the Faculty of Mechanical Engineering, University of Belgrade, Serbia. Her major fields of professional interest include: cognitive semantics, polysemy, critical discourse analysis, and meaning construction. She has authored several articles and chapters in the field of semantics and contrastive analysis.

Danijela D. Đorđević has been teaching English at the Faculty of Agriculture, University of Belgrade since 2009. She holds a PhD from the University of Belgrade – Faculty of Philology. Her main interests include English for Specific Purposes, Pragmatics, Semantics, Applied Linguistics and Terminology.
